# “Better than having no evaluation done”: a pilot project to conduct remote asylum evaluations for clients in a migrant encampment in Mexico

**DOI:** 10.1186/s12913-021-06539-5

**Published:** 2021-05-26

**Authors:** Ranit Mishori, Kathryn Hampton, Hajar Habbach, Elsa Raker, Anjali Niyogi, Dona Murphey

**Affiliations:** 1 Global Health Initiatives, Department of Family Medicine, Georgetown University School of Medicine, Washington, DC 20007 USA; 2 Physicians for Human Rights, New York, NY USA; 3 John W. Deming Department of Medicine, Tulane University School of Medicine, New Orleans, LA USA; 4 Department of Pediatrics, Tulane University School of Medicine, New Orleans, LA USA; 5 Medical Initiatives, Project Lifeline, Houston, TX USA

**Keywords:** Asylum-seekers, Telehealth, Immigration, Forced migrants, Medical-legal, Asylum, Evaluation

## Abstract

**Background:**

Asylum evaluations are highly specialized medico-legal encounters to collect physical or mental health evidence for use in immigration proceedings. Although the field of asylum medicine is growing, access to these evaluations is still inadequate, particularly for those in United States immigration detention or other forms of custody, such as under the U.S. Migrant Protection Protocols or “Remain in Mexico” policy. Given advances in telehealth in recent years and growing evidence of similar outcomes with in-person management, it seems prudent to examine whether remote modalities may also be effective for conducting mental health asylum evaluations in hard-to-reach populations.

**Methods:**

We analyzed the responses of 12 U.S. clinicians who conducted 25 cross-border remote mental health evaluations with clients in Mexico prior to the COVID-19 pandemic, and completed a post-evaluation survey regarding their impressions and experiences of the remote encounter. Data were coded through a process of thematic analysis.

**Results:**

The average evaluation time was 2.3 h, slightly shorter than might be expected from an in-person encounter. Five themes emerged from the coding process: rapport building, achieving overall goal, comparison of in-person vs. remote, technical issues, and coordination. Clinicians encountered a number of challenges including technical difficulties and a decreased ability to establish rapport. Nearly uniformly, however, clinicians noted that despite difficulties, they were able achieve the goals of the evaluation, including rapport building and diagnosis.

**Conclusion:**

Remote evaluations appear to achieve their intended goal and may be useful in expanding legal options for hard-to-reach asylum seekers.

## Introduction

Even before the COVID-19 pandemic, telepsychiatry and remote telehealth services have been an expanding field of practice in response to inadequate access and insufficient mental health workforce in rural areas of the United States. There is considerable evidence showing similar outcomes (diagnostic accuracy, care quality, efficacy, patient satisfaction) between in-person and tele-mental health services in the general population [1]. A systematic review of 452 published research articles on telepsychiatry found that treatment outcomes were similar to in-person sessions and that both patients and providers felt satisfied with services [2].

Recently, researchers have begun looking at the use of telepsychiatry for the purpose of conducting asylum evaluations in settings where in-person encounters are unavailable. Asylum evaluations are highly specialized medico-legal encounters whose purpose is to assess asylum seekers’ claims of persecution, torture or ill-treatment as part of a process to change their legal status for residence in a host country. Asylum evaluations can focus on physical evidence collection or on mental health evidence collection (in the form of psychiatric signs, symptoms or diagnoses related to the alleged trauma), or both. There are limited data from over 2 decades ago (when asylum grant rates were more favorable in general) showing that nearly 89% of those who had representation and received a medical evaluation gained asylum compared to the national average of 37.5% [3].

In Fiscal Year 2018, the most recent year for which we have complete data, 38,687 individuals were granted asylum in the United States [4] with 325,514 cases pending by December [5]. In 2019, just 31% of asylum seekers were granted asylum; grant rates vary greatly depending on the jurisdiction, individual judge, country of origin and whether or not the asylum seeker has legal representation [6].

Most asylum evaluations take place in the communities where asylum seekers reside while awaiting their immigration proceedings. However, some have to be conducted in detention facilities, which are often located in remote locations. Clinicians must travel several hours to perform exams, and the exams themselves can last several hours each, making the process of conducting asylum evaluations unfeasible for busy healthcare providers.

Since the implementation of the Migrant Protection Protocols (MPP, also referred to as “Remain in Mexico”), which has required asylum seekers to wait in Mexico during the duration of their immigration proceedings and to attend their hearings in courts along the southern US border, assessments have been complicated by the need to cross an international border to reach the clients and by physically insecure conditions in the open air encampments and informal shelters in Mexico where the asylum seekers are living [7].

The option to conduct an evaluation remotely enables clinicians and legal representatives to provide such services to clients who otherwise would not have access to an expert evaluation. Telephonic mental health evaluations, in particular, offer convenience, safety, and low cost for clinicians in comparison with paying and taking the time off for travel to those locations.

A recent comparison of 10 telephonic mental health asylum evaluations with 20 randomly selected in-person asylum evaluations found that telephonic and in-person evaluations were equally efficacious in over 26 clinically relevant areas, including obtaining a history of torture, psychiatric history, and reaching diagnoses [8]. Clinicians performing telephonic mental health evaluations stated that they did not find a difference in their ability to accurately diagnose in comparison with in-person evaluations, which required the same clinical standards and skills.

At the same time, the clinicians reported some challenges building rapport with the client without access to nonverbal information conveyed through body language and facial expressions. They found that checklists and cognitive tests were logistically more challenging to conduct over the phone; they also found that the mental status exam was less comprehensive, since they could not observe the clients’ motor activity, appearance, and facial expressions [8]. Another, albeit preliminary, study [9], reviewing 15 telephonic evaluations, reported that clinicians expressed increased comfort with telephonic evaluations following specific training.

In December 2019, Physicians for Human Rights launched a collaborative pilot project offering remote mental health tele-evaluations -- with video-conferencing options -- to asylum seekers residing in an open-air encampment across the US-Mexico border, and set out to assess participating clinicians’ perceptions of the experience.

### Project description

Physicians for Human Rights (PHR) -- a global non-governmental organization and a leader in capacity building for asylum evaluation programming and service provision -- launched the pilot program at the migrant encampment in Matamoros, across the US-Mexico border from Brownsville, Texas. As many as 3000 asylum seekers have lived in the encampment at one time since July 2019, when the US government began implementing the Migrant Protection Protocol (MPP) policy in that sector of the border [10].

More than 62,000 asylum seekers have been returned to Mexico under the Migrant Protection Protocols or Remain in Mexico policy, requiring them to wait for months for intermittent U.S. immigration court dates. Less than 1% of asylum seekers are granted the chance to exit the program and less than 5% have legal counsel [11]. Many of the clients in Matamoros do not have access to medico-legal asylum evaluations or related declarations, which could bolster their cases. The pilot was a partnership of several non-profit civil-society organizations. PHR recruited experienced asylum evaluators, Lawyers for Good Government^1^ (Proyecto Corazón), Pro Bono Asylum Representation Project (ProBAR),^2^ Justice For Our Neighbors,^3^ and local immigration attorneys facilitated the client referrals, and Project Lifeline^4^ assisted with client intake, coordinated scheduling, interpretation, and delivery of evaluations to legal organizations or specific attorneys for reconciliation of medical and legal documents.

## Methodology

### Protocol and setup

The pilot project provided both in-person and remote forensic and psychological evaluations and interpretation. A Project Lifeline intern coordinated all parties remotely and was physically present on site at the Resource Center Matamoros (RCM), where they could ensure connectivity between clinician, interpreter, and asylum-seeker, and that the privacy and confidentiality of the virtual encounters were established and maintained. The RCM provided private rooms and wireless internet access to support video tele-conferencing between the clinician, interpreter, and asylum-seeker via Skype or Whatsapp. At times, remote interpreters connected with the clinician and asylum-seeker, who were at the RCM for an in-person evaluation. Each encounter lasted between 2 and 4 h.

### Clinician preparation

PHR recruited experienced PHR-trained asylum evaluators via email request. Each evaluator was given a tip sheet with guidance on best practices for remote evaluations, a summary of the unique legal frameworks and an information sheet describing the local set-up. Project Lifeline coordinated with local attorneys and their clients, scheduled the evaluations, answered clinician questions before and after the interview, and connected clinicians to attorneys to deliver final reports to be reconciled with the asylum application.

A post-evaluation survey, developed by the project team, was emailed to the participating clinicians who conducted evaluations between December 14, 2019 to March 30, 2020, after their first remote evaluation to assess their overall experience during the pilot. The researchers’ detailed knowledge and experience with conducting asylum evaluations informed the set of questions.

The post-evaluation form included five open ended questions: 1. What is your overall impression of how the encounter went? 2. What were some challenges? 3. What went well? 4. How would you compare this encounter to an in-person evaluation? (For example, comment on your ability to collect the needed information, building rapport with the client, being able to assess their behavior, being able to assess for a psychiatric diagnosis, being able to assess credibility and malingering, etc.) 5. In your opinion, is this an acceptable way of conducting asylum evaluations for hard-to-reach populations?

We used a content analysis methodology to assess the responses [12]. Data were coded through a process of thematic analysis, starting with familiarization, then moving to coding, and then generating, reviewing and naming themes [13].

We coded content from the responses, as written by the clinicians performing the evaluations through open coding (creating tentative labels) and selective coding (comparing all the codes to the core question).

Two team members reviewed the answers and extracted themes and sentiments from the written text. Four team members reviewed the data and participated in an informal intercoder agreement process, where coders independently evaluate the data to check whether they will reach the same conclusions, to ensure best practices for data analysis, and to check the consistency of coding.

The project was deemed exempt by the Georgetown University School of Medicine (IRB # STUDY00001833) and was approved by the Physicians for Human Rights internal ERB.

## Results

We received feedback from 12 clinicians who conducted 25 remote evaluations from 14 December 2019 to March 30, 2020. The total number of clinicians who completed remote cross-border evaluations for PHR in that timeframe was 13; one clinician who had completed one evaluation did not return the feedback form. All were psychological evaluations, except for one evaluation which was combined physical and psychological. All clinicians were experienced in conducting in person asylum evaluations; three had also completed evaluations in Matamoros in person. Clinicians included psychiatrists (5; 2 of which were child psychiatrists), internal medicine & pediatrics (1), pediatrician (1), psychologists (3) and clinical psychologists/ neuropsychologists (2). During the majority of evaluations, the clients were in the Resource Center Matamoros, though a few were in their hotel rooms or apartments where they lived. Evaluation time ranged from 70 min to almost 4 h, with the average evaluation time being 2.3 h. All but one of the evaluations required an interpreter.

We identified five domains within the feedback provided by the clinicians: rapport building, achieving overall goal, comparison of in-person vs. remote, technical issues, coordination.

Overall, the clinicians encountered a number of challenges with remote evaluations in this context, but as one evaluator stated, it is “.... certainly better than having no evaluation done”. Similar comments included: “Even though it isn’t ideal, I think it’s an acceptable way for asylum seekers to get the help they need”, “It allows us to reach many more folks”, “I would still do it again, because I think the exam is more important to have than no exam at all.... But in person is much better.”

Clinicians observed a decreased ability to establish rapport with the clients, technical difficulties that affected the encounter, and diagnostic challenges being unable to visually assess reactions. They also stated the importance of coordination and interpretation. Nearly uniformly, this select group of clinicians noted that despite difficulties, they were able achieve the goals of the evaluation.

Table 1 summarizes the various aspects of the evaluation and provides illustrative quotes from clinicians about their experiences.

**Table 1:** Clinicians’ Experiences Conducting Remote Asylum Evaluations. (In parentheses are the specialty of the evaluator and the number of remote evaluations conducted as part of this pilot project)

Domain	Positive or Neutral Sentiment	Negative Sentiment
Connecting with the client and building rapport	Rapport was easy to establish I thought, I think we connected. (psychologist, 1 evaluation)	My efforts to make the connection were exhausting, and I was left feeling generally tired and less than satisfied after 3–4 h of hard work. Building rapport is certainly more challenging especially since your trying to collect information that is very sensitive and painful (neuropsychologist, 12 evaluations) [In the in-person evaluation it] is much easier to establish rapport, easier for me to allow time for the client to reflect, take a step back emotionally as needed (med-peds, 2 evaluations) I was not able to comfort him well without being there in person… It felt very awkward over the computer and I was not able to establish the kind of connection that I am able to create when I do these exams in person. (pediatrician, 1 evaluation)
**Achieving the goals and objectives of the encounter**	I was able to gather sufficient information from the clients about their pre-immigration histories, experiences of migration, current symptoms and circumstances (psychologist, 1 evaluation) The encounter overall was successful, and the client was granted asylum (med-peds, 2 evaluations) Overall, considering the circumstances, one is able to get the work completed. (psychiatrist, 1 evaluation) I was glad that I was able to perform a full physical exam and psych eval without needing to travel to Matamoros. That made it very convenient and I was gratified to be able to do this. (pediatrician, 1 evaluation) It is ideal when an evaluation is conducted in-person. However, considering that that is not always feasible, this is a good alternative. (psychiatrist, 2 evaluations)	While I was able to technically complete the exam, it was not the same kind of evaluation as my usual ones. I got them done despite the conditions. (neuropsychologist, 12 evaluations)
**Comparing Remote vs. in-person evaluations**	It felt very similar to in person assessments I have done. (child psychiatrist, 1 evaluation) Very comparable—it really was not very different from meeting in person, to my mind. (psychiatrist, 1 evaluation) [No challenges] because of remote nature of the interview— but many of the usual challenges of assisting client to focus his/her story (psychiatrist, 1 evaluation) I worked extremely hard to try to put more expression than usual into my voice, and to compensate for the loss of verbal and physical connection. (neuropsychologist, 12 evaluations) I was comfortable with the remote format and felt well-equipped to establish rapport with the clients and gather necessary clinical information (psychiatrist, 2 evaluations) I think I was able to assess for the psych diagnosis and assess for credibility, etc. fine. (pediatrician, 1 evaluation) I did not feel this significantly altered my ability to make a diagnosis, however, nor to comment on the client’s credibility (med-peds, 2 evaluations)	With clients who are more reserved, this mode of conducting an evaluation would be more problematic. (psychiatrist, 1 evaluation) When I do interviews in person, I can make a full assessment and diagnosis in a conversational manner. In contrast, too often with the remote interviews, I had to go through a checklist of symptoms with the person. Furthermore, I could not make visual observations—of dress, grooming, general physical appearance, bodily movements. I could not observe hyperactivity, tics, or abnormal movements. I could not see the condition of their skin, nails or hair. I could not see body type or weight. (neuropsychologist, 12 evaluations) I definitely prefer in-person interviews if possible. (med-peds, 2 evaluations) I was not able to observe the client’s gait. Due to being on-screen sometimes full facial expressions were not fully visible which was challenging in doing a mental health evaluation.(forensic psychiatrist, 1 evaluation) The distance reinforced the avoidance aspects of her defenses as it was far more difficult for her to bear the pain of describing some of her experience (child psychiatrist, 2 evaluations) It was also much harder to make a psychiatric diagnosis (neuropsychologist, 12 evaluations)
**Set up, technical Issues and their impact on the evaluation**	There were several moments in which the interpreter lost her connection to the online videoconferencing site we were using (Skype). However, this technical glitch created only short (2–3 min) delays with minimal discernible impact on the flow of the assessment. (psychiatrist, 2 evaluations)	Given the couple of connectivity issues, it was difficult to return immediately to the same thread we were on before. I did not feel this significantly altered my ability to make a diagnosis, however, nor to comment on the client’s credibility. (med-peds, 2 evaluations) It is also harder to assess things such as affect of the individual due to poor connection (psychiatrist, 1 evaluation) We worked around [the audio] issues by repeating things that were sometimes lost (forensic psychiatrist, 1 evaluation) Lower video quality made it more difficult to pick up on non-verbal cues. For example, it took longer than usual for me to realize the client’s father was becoming emotional. (child psychiatrist, 1 evaluation) In one instance, the phone connection was so poor, and the client was so frustrated, that she was becoming upset and I chose to stop. (neuropsychologist, 12 evaluations) Several times the communication either got stalled or completely disconnected. Had to call back and forth 2 times. (psychiatrist, 1 evaluation) Having 3 people in 3 different locations on this particular platform was not ideal, especially when dealing with highly sensitive and emotion-laden information. (child psychiatrist, 1 evaluation) Difficulty making the initial connection; losing the connection repeatedly and calling back; poor signal, difficulty hearing the client. I was glad that I had a video connection for at least a few minutes, because I could see where they were: on the street, or in the doorway of a store. Neither of them had told me that they were outside. (neuropsychologist, 12 evaluations) The legal representative did not set up the interview or do introductions, so it was an awkward start. [He stayed] in the room, which made the client even more reticent (psychologist, 2 evaluations)
**Coordination**	The scheduler was there on time and stayed to ensure that things were properly connected. (child psychiatrist, 2 evaluations) It was well organized and timely; I was able to contact the liaison and the attorney to get additional information that was required (Psychiatrist, 1 evaluation) When I was not able to see the scars well, [coordinator] sent me close up photos on WhatsApp so that I could see better. (pediatrician, 1 evaluation) If neither interviewer nor interpreter could be in the same room with her, it might have been useful for her to have another support person present. (child psychiatrist, 2 evaluations)	The client actually started crying, and then I asked [coordinator] to please get him a tissue. If I were there, I would have put my arm around the guy. (pediatrician, 1 evaluation)

## Discussion

Despite multiple challenges, and while perceived as less ideal than in-person evaluations, clinicians felt that remote evaluations -- even across international borders and in an unstable setting -- achieved their intended goals and were “better than having no evaluation done” at all. This is the first published article, to our knowledge, to involve or report on audio and video-conferencing remote asylum evaluations, and to involve clients outside of the US, across international borders and residing in unstable housing in a migrant encampment.

Overall, the 12 clinicians conducting 25 evaluations did not feel that the challenges inherent in the remote aspect of the encounter or the video-conferencing technology prevented them from achieving their intended goals of providing this critical medico-legal service to asylum seekers. In post-evaluation feedback they reported that rapport building and diagnostic accuracy did not suffer significantly, in a way that would prevent them from fulfilling the evaluation’s objectives.

Beyond the clinicians’ perspectives, program staff encountered several challenges related to coordination among stakeholders. The unstable conditions in this transient setting and the ever-changing legal and physical environment have proven to be major challenges to various aspects of the project. However, given the low rates of medico-legal support provided to this population, even doing a few evaluations could potentially affect the lives of individuals and families otherwise not afforded this opportunity.

Our pilot study and conclusions are limited by a variety of factors. First, this pilot was conducted at a single remote location, which may differ in significant ways from other settings with different resources, partners, or technical infrastructure, so we cannot generalize. Second, the clinicians were a pre-selected group of highly experienced individuals whose expertise with this population may have been enough to overcome challenges rendered by the remote nature of the encounter. It is unclear whether less experienced clinicians would feel that they could achieve the encounter’s goals given the circumstances. Third, methodologically, the feedback format involved open-text written answers, which may not capture the full scope of the clinicians’ experience (for example, as might happen during an in-depth interview), and, fourth, some quotes were taken from clinicians who conducted multiple remote evaluations, so their comments may be over-represented in our analysis. Additionally, this pilot focused on remote mental health evaluations. Physical evaluations might present additional challenges due to the need to assess physical signs (such as scars), which may be more difficult to evaluate via remote technology, or in public spaces. Importantly, we did not seek feedback on the process from asylum seekers themselves, so we do not know the extent to which this format was acceptable to them.

This pilot was conducted prior to the COVID-19 pandemic, which forced when many health professionals to pivot to remote patient encounters as part of the daily work. It is likely that at the time of this pilot, clinicians were not as familiar with conducting remote encounters and assessments. Were this project conducted later, clinicians would have possibly felt more at ease with the technology and and other challenges of remote assessments.

Critically, it is unclear how the affidavits produced as part of the remote medico-legal evaluations were perceived by U.S asylum adjudicators or immigration courts. Did they view the assessment of the clients’ credibility differently? Are there regulatory barriers to conducting an assessment with a client who is not only in a different state, but in a different country? To date, we have received no indication that they were not admitted as parts of the case materials. We also do not know whether and how the resulting affidavits and declarations have been used in the legal proceedings of the clients, or what the case outcomes are.

The acceptance of remote evaluations – especially those conducted in other countries -- in immigration courts as equal or nearly-equal means of clinician assessment may potentially have domestic and global ramifications. Asylum evaluations are highly relevant for asylum and immigration proceedings in all refugee-receiving countries and in fact such evaluations are being conducted, and their impact and effectiveness studied, in multiple countries, including the UK [14], Italy [15], and the Netherlands [16]. The international standard used as a reference for asylum evaluations around the world is the UN Manual on the Effective Investigation and Documentation of Torture and Other Cruel, Inhuman or Degrading Treatment or Punishment, also known as the “Istanbul Protocol” (IP) [17]. The IP -- which is the PHR standard for the evaluations -- outlines objective criteria for evaluation, write-up and assessment of torture documentation and state obligations to promote access to objective medical evidence of torture. The UK Supreme Court has affirmed the probative value of evidence collected according to Istanbul Protocol standards in asylum cases [18]. Evidence used from a study such as ours may enable clinicians in other parts of the world to advocate for the use of remote evaluations to assess hard-to-reach torture survivors and asylum seekers, across borders, in remote areas, or in detention. Evidence of the utility of such encounters may support experienced evaluators in conducting remote assessments in countries and settings where medico-legal experts are hard to find, as part of international justice and accountability mechanisms.

Beyond their role in legal proceedings, remote assessments have also been used for advocacy purposes, for example, to force governments to transfer asylum seekers in Australia [19], and in PHR’s own advocacy activities, such as in a 2021 project titled “Forced into Danger” where we conducted an analysis of 95 medico-legal affidavits, most of which were obtained remotely, to document human rights violations of migrants in Mexico, resulting from the US Migrant Protection Protocols [20].

## Conclusion

Asylum evaluations conducted remotely, even across international borders, are acceptable to the clinicians conducting them for the purpose of assessing forced migrants’ psychological status as part of their legal proceedings. Remote evaluations may be useful in expanding legal options for hard-to-reach asylum seekers.

As the world adjusts to incorporating remote activities in multiple domains of life (including health, education and legal services) as a result of the COVID-19 pandemic, we remain hopeful that remote evaluations will be accepted as a routine form of medico-legal service to the benefit of isolated or hard-to-reach communities. As one of the clinicians noted: “I believe we should consider videoconferencing-based evaluations as likely an increasingly valuable and even indispensable tool in the evaluation of asylum seekers and other global mental health endeavors”.

## Data Availability

The datasets used and/or analysed during the current study are available from the corresponding author on reasonable request.

## Footnotes

1 https://www.lawyersforgoodgovernment.org/

2 https://www.americanbar.org/groups/public_interest/immigration/projects_initiatives/south_texas_pro_bono_asylum_representation_project_probar/

3 https://njfon.org/

4 https://projectlifeline.us/
